# Operators’ good community engagement practices in energy projects: examples from the UK deep geothermal sector

**DOI:** 10.1186/s13705-026-00566-y

**Published:** 2026-02-19

**Authors:** Stacia Ryder, Melanie Rohse, Corinna Abesser

**Affiliations:** 1 Department of Sociology and Anthropology, Utah State University, 0730 Old Main Hill, Logan, UT USA; 2 Department of Geography, University of Exeter, Stocker Road, Exeter, EX4 4PY UK; 3 Global Sustainability Institute, Anglia Ruskin University, East Road, Cambridge, CB1 1PT UK; 4 British Geological Survey, Keyworth, Nottingham, NG12 5GG UK

**Keywords:** Public engagement, Operator rationale and motivations, Ethics of care, Trust, Social acceptance, Public participation, Subsurface, Ethos, Values

## Abstract

**Background:**

To be fair and inclusive, the transition to net zero must combine technological rollout with enabling communities to participate in energy decision making. Participation in the energy system can take many forms, and one area with great potential for improving fairness and inclusivity is the siting and implementation of renewable energy infrastructure. In the UK, one emerging renewable energy technology is geothermal energy. Our study aims to understand how the geothermal industry is approaching engagement in a country with limited geothermal development and research into engagement practices.

**Results:**

Using qualitative interviews conducted at three geothermal energy sites in the UK, we reveal that the operators interviewed appear to share an ethos characterised by honesty, trust and relationship-building. This ethos underpins good community engagement practices, such as approachability, accessibility, flexibility and two-way communication. We also observe that the operators are proactive in their engagement activities and responsive to queries from local communities.

**Conclusions:**

Our results provide an initial analysis of engagement practices in the UK geothermal industry and offer a model of good community engagement practice around geothermal energy. Guided by an ethics of care towards communities, operators can routinely go beyond the minimal engagement requirements of planning. This enables them to address communities’ concerns, act on them, and maintain a dialogue between different stakeholders. There is a need for policy instruments to support this approach and establish higher engagement requirements for energy projects.

**Supplementary Information:**

The online version contains supplementary material available at 10.1186/s13705-026-00566-y.

## Background

As energy demands increase worldwide, the UK is facing both an energy and a climate crisis. This necessitates a rapid transformation of the UK energy system, which can be achieved in part through the pursuit of a clean energy transition [1]. With nearly half of the UK’s energy being used for heating, decarbonising this system is essential [2]. For the UK to move away from fossil fuels and achieve net zero by 2050, the nation’s energy portfolio needs to be diversified.

Using deep geothermal energy is one possible approach to achieving long-term energy security and reducing carbon emissions [3], particularly with regard to decarbonising building heating and cooling systems [4]. While the UK is not currently considered a country with geothermal capabilities, geothermal energy could contribute considerably to meeting local and regional heat demands in several regions [5–7].

The exploration and exploitation of geothermal energy is intrinsically technical, yet geothermal developments also include social, spatial and political aspects that have so far been neglected in geothermal energy research [8]. There is low public awareness of geothermal technologies in general, and societal engagement is predominantly neglected or typified by one-way information sharing with the goal of increasing social acceptance [9]. However, there are some notable exceptions to this, for instance in Iceland where the geothermal sector has managed to foster trust beyond specific projects through societal engagement and has established a ‘Social License to Operate’ [10]. In Spain, a group of residents initiated a bottom-up geothermal project to move away from fossil fuel sources of heating [11], thus demonstrating the potential for collaboration between communities and operators.

The situation in the UK mirrors the dominant trends outlined above. Two recent studies focusing on the UK [12, 13] have shown low public awareness of geothermal energy technologies. Regarding societal engagement, UK geothermal projects have not yet engaged communities through bottom-up processes, and engagement strategies have tended to be focused on reducing contention around specific projects [14]. Assuming that geothermal energy could play an important role in helping the UK meet its Net Zero commitments [15], several geothermal energy projects are at different stages of conceptualisation and development, including some operational schemes [7]. Yet, operators’ practices of engagement are under-researched, and it is unclear which good ones have been established and where improvement is needed.

Recognition of the direct connections between community engagement, public support, and policy decisions across different energy sectors is crucial for all technologies, including renewables. How public and community concerns are addressed may determine the balance between public support and opposition. This article adds to existing literature on engagement by examining the values and attitudes of operators that underpin an ethos of engagement, and how this shapes their approaches to addressing public concerns.

We use qualitative research to explore perceptions and experiences of community engagement at three deep geothermal energy sites in the UK. We aim to examine under-explored factors that shape the design and implementation of community engagement efforts, and how communities perceive these efforts. Our research questions are:

1. What types of actors lead the engagement efforts of UK geothermal operators^1^?
2. What values and attitudes towards community engagement do operators hold?
3. What is the role of operator attitudes and values in shaping community engagement strategies across the UK geothermal landscape?

This article is divided into five sections. First, in the remainder of this background section, we give a brief overview of the existing literature on public and community engagement, including in geothermal energy development. Second, we describe our methods of data collection and analysis. Third, the results section is split into three sub-sections, which explore (1) the types of actors that lead engagement in geothermal projects and their perceived effectiveness; (2) what values and attitudes underpin operators’ engagement ethos; and (3) how this ethos connects with designing engagement. Fourth, we discuss our findings, consider their implications for policy, and highlight some limitations to this study. We end with a brief conclusion section that summarises our key arguments and considers opportunities for future work.

### Overview of literature on public and community engagement in energy developments, including subsurface projects

Community engagement has increasingly been integrated into resource governance and energy development for several decades [16–18], recognising the need to establish energy democracy. Research on community engagement has identified several factors that have an impact on good practice, including who is involved, why and how they are involved (Table 1).

**Table 1:** Factors influencing community engagement

Factor	Explanation	Example of studies identifying this factor
Who	What actors are leading the engagement? Which ones are/should be included?	[19–21]
Why	What are the actors’ rationales for participating in the process?	[22–24]
How	1) How are engagement and participation opportunities structured? 2) In which direction(s) does information flow between actors? 3) What is the extent of public control and how authentic is the engagement?	1) [25, 26] 2) [27] 3) [19, 27, 28]

There is a body of literature focusing on community experiences of engagement in energy projects, especially with a focus on wind energy [29, 30]. Recently, though, there has been a greater focus on the role the operators of a technology play in engagement, in terms of how they view the public and how they think about engagement [23, 31]. However, the role of their ethics and values in shaping their approaches to the public and to engagement remains under-examined.

What constitutes ‘effective’ engagement is contextual. Transparency, inclusivity, justice and fairness have been identified as good decision-making practices. Furthermore, engagement should be unconditional and the decision-making process allow sufficient time for all stakeholders to get involved [32–34]. However, examples of ineffective engagement – where processes are unfair, conditional, insufficient, opaque, and seen as inauthentic – abound in past energy projects [35–37]. Some contemporary studies of community engagement have revealed that there remains a high level of distrust in operators [18, 37–39].

Public perception and experience of energy technologies are increasingly important in energy policy contexts. Indeed, they can influence whether individual projects, or even entire industries, move forward, and the speed at which they do [40–42]. For example, research on community experiences and public perception of hydraulic fracturing for shale gas suggest that the practice faced continuous opposition not only due to induced seismicity risks, but also, crucially, due to failed community engagement and distrust [37, 43, 44]. This was compounded by the UK government’s narrow view of community engagement as a road to social acceptance of a technology, which failed to capture and address the wide-ranging concerns and risk responses of the public to shale gas, including issues of justice and equity [45].

A driving factor in community engagement around energy projects has been the desire to reduce conflicts arising from communities’ opposition to a project. Community opposition tends to be framed as a ‘barrier’ to the progress of proposed projects, which leads to an often narrow and instrumental engagement, aimed at securing public and local approval for a project to move forward, what is termed here ‘social acceptance’. Research findings about the effectiveness of this ‘social acceptance’ approach are mixed [42, 46–50]. Several researchers criticise this approach, exposing how it is underpinned by operators’ and policymakers’ reliance on specific visions of ‘imagined publics,’ where publics are thought of as lacking adequate information. Previous research has frequently revealed negative attitudes of operators towards the communities hosting their energy infrastructure. This includes the notion that they are uneducated and uninformed, or merely a social risk or barrier to a project’s development, often assuming that they are only objecting to a project because it is on their doorstep (despite over a decade of research refuting NIMBYism in energy contexts, see [29, 46]). These visions have an impact on engagement design, which becomes guided by the belief that ‘imagined publics’ can be persuaded to back a technology through the provision of information, with the assumption that information will lead to increased understanding and therefore acceptance [51, 52]. As a result, communities often report feeling disrespected and ignored, and view the engagement activities that are done as a tick-box exercise [53]. From a justice perspective, focussing only on social acceptance elides the important aspects of process and procedure in decisions about energy projects. This suggests the need to move beyond ‘social acceptance’ to understand what communities expect from engagement [44] and the plurality of community responses to proposed energy projects, from support, to opposition, and even ambivalence [54–56].

## Methods

### Description of project sites and communities

While there is a history of geothermal underground exploration in the UK, the pursuit of deep geothermal energy is still nascent. According to a 2023 report [7], over 20 projects are at pre-feasibility and feasibility stage, with many on hold for various reasons. Several projects are under development and only a handful are operational. This guided our case selection. According to good practice, community engagement should start early and be continuous, so we sought three cases at different stages of development to be able to probe participants both on current and future engagement plans and seek their reflections on past engagement. This would also allow discussion with community members both on experiences of engagement and on expectations regarding good practices of engagement. As part of our ethical commitment to the participants, especially considering the small size of the geothermal industry in the UK, we have anonymised the locations of the sites and we can only give brief descriptions of those locations within the boundaries of our ethics approval.

The first and second site (locations 1 and 2) are in rural areas with a history of mining, with the nearest small town in each case around five miles away. One site is operational while the other is under development. The third site is in an urban area (location 3), in a city with a population of around 250,000. The area has an industrial past, including a long history of mining. The proposed geothermal development is located on an existing industrial site. The project has received planning permission but was not underway at the time of the research.

### Data collection and analysis

This study started while COVID19 pandemic restrictions were still in place, and the research team opted to start with interviews online. When the restrictions were eased, two site visits were organised. As a result, the data collection was spread from July 2021 to June 2022. In that time, the research team conducted 23 semi-structured interviews with key government, industry, and community stakeholders (see Interview Guide in Supplementary Material). This included individual and group interviews (up to three participants at a time), with a total of 30 participants. Given the COVID19 pandemic restrictions, 24 participants took part virtually, and six took part face-to-face during site visits to location 3 (January 25–28 2022) and locations 1 and 2 (June 13–16 2022). Semi-structured interviews are a common qualitative research method useful for developing an in-depth understanding of people’s views, experiences, beliefs and perceptions [57]. They thus were appropriate for our objective of investigating operators’ and communities’ views and experiences of community engagement in geothermal projects.

While this article focuses on operators’ practices of community engagement, the study also had the goal of understanding the regulatory frameworks governing geothermal energy developments in the UK. This is reflected in the recruitment of participants. In a first phase, the interviews focused on these frameworks, including questions of community engagement. In a second phase, they focused on the operators’ and communities’ experiences at specific sites. As a result, the recruitment criteria were different (but overlapping to an extent) for phase one and two. In phase one, we sought to speak with experts in regulations of the UK geothermal energy sector, be they operators and industry organisations, local authority planners and representatives, or representatives from regulatory bodies at the central level. To recruit participants, we engaged in purposive sampling and snowball sampling [58, 59]. Initial interviewees were first identified through Abesser’s professional network and recruited via email. Further participants were identified by these initial interviews. In total, ten interviewees took part in this phase between July and December 2021. Owing to the small size of the industry, six participants had previous or existing links to one or several of the site locations (even though this was not a recruitment criterion for this phase).

In phase two, we sought to speak with representatives of industry organisations as well as of local authorities and communities with direct experience of a geothermal energy project, regardless of the stage of development of the project. After identifying three suitable locations, in-person recruitment and snowball sampling were used to reach out to community members. We knocked on doors of residents in the closest proximity to the site at location 3, leaving recruitment flyers. At all locations, we contacted community organisations that would be considered potential stakeholders, including via social media. At location 1, we advertised two focus groups, though ultimately these resulted in interviews.

Of the thirty participants, ten represented energy industries, four were local planning authority representatives, four represented national regulatory bodies, and 12 were community members (including local government representatives) from nearby the sites. Table 2 summarises the spread of participants across the sites, reflecting that some of them had connections to more than one site, but also that some had no connection with any of the sites. Men are overrepresented, making up 70% of our interviewees, however, this reflects the degree to which the energy sector is male-dominated. During the interviews, community residents were asked specific questions about their knowledge and support of local geothermal energy projects, their experiences of community engagement around the project, and what risks or benefits they saw. Operators and local governments were further asked about previous efforts and plans for community engagement as well as their ideas around best practices for regulating geothermal energy in the UK.

**Table 2:** Spread of participants across sites

Location	Non-location specific	Location 1	Location 2	Location 3
Number of participants	8	10	6	7

The interviews were recorded and professionally transcribed. The transcriptions were analysed using NVivo software, following an iterative process. All the authors familiarised themselves with the data by reading through the transcripts, thus preparing for a thematic analysis [60]. In early analysis, memos were developed and an initial inductive coding guided the research. In this first phase, 33 inductive codes were defined. At this stage, they were kept descriptive, using terms such as ‘project background’, ‘energy independence’, ‘engagement champion’, or ‘perceptions of the public’. We wrote and recorded field notes during site visits, which allowed us to triangulate our interview data [61]. We reviewed the field notes iteratively throughout the analysis to ensure the validity of the coding scheme. Combining these methods helped to inform a deeper understanding of the particularities of each geothermal project.

In a second phase of analysis, the codes were grouped within themes [57] reflecting the objectives of the study to explore questions of regulation, community engagement practices, and risk perceptions (see Table 3). In this process, not all descriptive codes were relevant, and we focused on the ones that best fitted our objectives (e.g., while the ‘project background’ code was useful to easily access contextual information about each site, it was not as relevant to the thematic analysis).

**Table 3:** Overarching themes and the codes that informed them

Policy and regulation	Operator engagement practices	Community perceptions of risks and benefits	Community experiences of engagement
• Barrier • Geothermal misunderstood • Policy problem or solution • Regulation other • Regulation scale • Regulation seismicity	• Bad actor • Community engagement • Engagement champion • Geothermal champion • Perceptions of public • Positive examples geothermal • Trust	• Benefits • Public perception • Risk of concern	• Community engagement • Engagement champion • Geothermal champion • Trust

We delved deeply into those themes by each taking notes on one given topic and meeting to discuss our observations and how the quotes within the themes related to the literature. The information within the themes was re-organised, moving away from the descriptive codes to create more analytical categories related to the literature. Regarding operator engagement practices, the topic of this article, the analytical categories were (1) the types of actors leading engagement strategies, (2) the attitudes and values of operators regarding communities and engagement, and (3) the design of good engagement practices. These analytical categories structure the results section of the article, and predominantly draw on operators’ views, which are supplemented with communities’ experiences where appropriate.

## Results

### Types of actors leading operator engagement and their perceived effectiveness

While much of the research on community engagement around energy infrastructure projects highlights the different types and strategies of engagement, less research has examined questions around the importance of *who –* including both individuals and/or institutional actors *–* is leading it. In US and Canadian shale contexts, operators often organise engagement through community liaison officers, or by outsourcing the work to public relations or media companies, and/or consultancies [62]. Yet, geothermal operators in this study do not view this as the most effective strategy. More than one operator suggested they wanted to be clear that their engagement efforts were not tied to a PR approach:

*“I’m not convinced that people are impressed by glossy PR materials. I think people are far more impressed if they raise a concern that you get back to them as a real human being who’s prepared to look them in the eye and answer their questions directly and sometimes say*,* ‘Look*,* we can’t guarantee that that won’t happen but we can tell you this and we will always be straightforward with you.’” – Interviewee 17 (operator)*.

Rather, operators highlighted that doing their own community engagement creates more opportunities for relationship building and connecting with concerned community members, in contrast to more prescribed approaches to engagement when hired PR companies focus on image building. Thus, having on-the-ground technical team members involved in engagement is perceived as a successful approach for interfacing with communities.

However, scientific and technical team members may often not have training or experience in science communication or community engagement, which can hinder their ability to effectively communicate the technical aspects of a project to non-experts. With this in mind, another operator emphasised the importance of hiring someone who had direct experience in communicating with communities on environmental issues more broadly, and how beneficial this has been.

Employing individuals who may be less familiar with the technical aspects of the project can help ensure layperson language is used in communication efforts:

*“Not coming from the engineering side… I could perhaps imagine what it was like to be a local resident a bit better and think*,* well actually*,* no*,* they’re not gonna understand that…So that was an advantage and the challenge was to try and communicate the science*,* but to be honest*,* in many ways it’s been less about the science and more about convincing people that we’re not trying to hide something really difficult.” – Interviewee 17 (operator)*.

The ability to put oneself in the shoes of residents prompted this interviewee to highlight the importance of making connections and empathising with residents’ concerns. In turn, local residents particularly appreciated the focus on making complex science accessible to local communities:

*“Don’t get too techy as it were but have…so have the really technical stuff for people that wanna bombard you with engineering questions and then have the idiot’s guide to how the system works*,* for everybody else. And that’s what they did.”* – *Interviewee 20 (local community)*.

Another operator pointed to how long-term investments in communities and having a company be comprised of local staff can also improve community perceptions of a company or project:

*“It helped that we live here*,* we weren’t somebody coming in from out country to do this on their patch*,* we’re [local] people on the staff and I’ve been here 40 years. We’ve got a long history of being*,* not in that exact community*,* but being nearby and making it evident that we had [the region’s] interest at heart. All that helps too.” – Interviewee 3 (operator)*.

In this case, the company is not only working in the area but also rooted locally over a long period of time. Operating as embedded local actors that have demonstrated long-term commitment to local workforce development, representatives of the geothermal industry interviewed tended to consciously move away from failed engagement practices deployed by other energy sectors. This effort focuses on the “who,” in terms of who comprises a company and represents that company in the community.

### Operator attitudes and values: developing a strong engagement ethos

This section focuses on the attitudes and values operators display towards local communities and engagement, which underpin their ethos of engagement. Multiple operator representatives highlighted the importance of approaching local communities with respect and honesty, focusing on trust-building:

*“If people believe that you’re telling them everything and telling them the truth*,* that goes an enormous way to making them feel more comfortable about what you’re doing. – Interviewee 3 (operator)*.

A genuine commitment to telling the truth moves away from focusing on the positives while glossing over points of concern. It comprises operator honesty about both technological and non-technical aspects of the project, including potential risks, benefits, unknowns, and uncertainties, and how risk might be mitigated:

*“We have always taken the stance that there’s no point in hiding stuff because it’s just gonna come back and bite you…so what we would try and focus on is*,* yeah*,* there is stuff we don’t know*,* but these are our systems in place and our control systems in place to make sure that that risk is mitigated.” – Interviewee 7 (operator)*.

Being honest about seismicity and risks – the most widely shared public concern according to existing research on deep underground energy interventions – was particularly important:

*“People don’t mind so much if you’ve told them [something] might happen. If you have said*,* ‘We’re gonna do this over the next month and we’re gonna be pumping this water round and you might feel or hear some little bumps. That’s what it is. It is us*,* don’t worry about it*,* it’s still going to be ten times too small to do even cosmetic damage to your house.’…there may still be some people who are worried but that’ll be a whole lot better than not saying anything until it happens and then explaining it away. That was always my view with do all this upfront and tell people upfront what might happen.” – Interviewee 3 (operator)*.

This commitment to being upfront about unknown and potential risks was valued by community members:

*“I think it’s honestly being upfront to people like…‘This is possibly…this could happen [or] this could happen [or] This won’t happen.’ Etc. But it is a new kind of industry so there’s always gonna be a possibility that something*,* like the small seismic event they had*,* might happen. But*,* when [a seismic event] did [happen]*,* they were upfront about it*,* they put it out on Facebook and on the website and within hours everybody knew what had happened and they were ringing me up first thing in the morning.”* – *Interviewee 20 (community member)*.

Operators, residents, and local council representatives across the sites spoke further about the importance of community engagement focused on trust building, which requires making long-term commitments and the investment of both time and effort to follow through:

*“They [the community] trust them [operator] when they put information out. And I think that’s what they’ve done really well*,* is build the trust in the community. They’ve made sure the local community have benefitted. When the drilling team have come in*,* they’ve stayed in local B&Bs*,* they’ve spent money in the local community. That makes the sell so much easier to a community*,* when they can see those benefits….Whereas I think if you’ve just got a development going on behind a fence that nobody knows what’s happening*,* what’s going on*,* and you get a few tweets about a rumbling*,* before you know it you’ve got lots and lots of bad publicity and one lot of bad publicity in one project can just kill all the rest.” – Interviewee 9 (local government)*.

The examples above demonstrate how a commitment to respect, honesty, and trust underpins these operators’ ethos of engagement, which emphasises the importance of relationships and dialogue with local communities. This was predominant across location 1 and 2, perhaps because of their more advanced stages. However, the picture was murkier in location 3, which was at an earlier stage of development. While the operators were able to share their experiences of engagement in past projects at other locations, little engagement had happened at location 3. In fact, the residents interviewed were uncertain about the exact nature of the proposed project, about its timeframes, and at times also about its location. They expressed distrust in the local authority, which they reported had failed on delivering past energy projects and which was perceived as reluctant to share updates on the geothermal project. This could cause issues further down the line, with one local resident noting: “because there hasn’t been much public engagement from the council, there is a lot of ignorance over what [the geothermal project] is and what it could mean” (Interviewee 14; community member). From this study, it is not possible to say what impact this will have on the proposed project. Yet, it shows the importance of developing an ethos of engagement early, especially one that focuses on relationships, to foster trust with communities.

### Connecting operator engagement ethos and good engagement practices

The ethos of the actors leading operator engagement shapes the engagement strategies pursued. In the case studies described, operator attitudes toward communities potentially affected by projects tend to be empathetic and understanding. This contributes to an ethos of engagement that emphasises respect, honesty, trust-building and relationships. In this section, we investigate how this approach is reflected in specific engagement actions that reflect the importance of the project leaders’ engagement ethos and attitudes toward communities.

#### Approachability, availability, and commitment to information sharing

The sharing and communication of information is a crucial part of community engagement. Here, the operators expressed a commitment to ensuring that information was accessible through multiple channels from early project stages, giving local communities as much information as possible from the beginning of a project. In one instance, an operator explained how this early and continuous effort at information sharing contributed to changing one resident’s perception of the project:

*“[We] literally love-bombed people with information. [A resident] was basically a bit anxious about it*,* but he turned into our biggest advocate*,* and he was all over Facebook going*,* ‘It’s a disgrace*,* it’s a disgrace*,* this bloody thing*,* they never tell us what’s going on …’ and he turned into our biggest advocate. He used to come down every day with his dog and talk to the engineers…he made us a huge great big photographic mosaic for us to use in our public meetings. It was amazing.” – Interviewee 16 (operator)*.

Early and continuous provision of information is an important part of good practices of engagement, even when some engagement events and strategies are geared toward one-way information sharing. But engagement goes beyond community access to information. There is also a strong desire from community members that the operator be accessible to them. For example, one issue raised in the UK shale context was the lack of ability to reach or get responses from operators [37]. In the case of geothermal energy, residents at locations 1 and 2 described the opposite:

*“[The operators] have been very good. They’re quite forward leaning…and totally willing to come and talk*,* which I think is good.” – Interviewee 24 (local government)*.

*“Once it was explained and shown how it worked*,* the village has been very positive actually*,* particularly the way that they kept folk involved …They’ve kept folk in touch with leaflets*,* posters*,* things happening on the Facebook page…There’s a community liaison person. She comes along to the village coffee mornings. So there’s an ability to speak to her…So yeah*,* they’ve definitely kept people in the loop with what’s going on… So they certainly made themselves noticed*,* ‘Yes*,* we’re approachable. You can come to us*,* you can question us on what we’re doing.’…People can see what’s happening*,* discuss it*,* debate it*,* say do we agree with it?…The fact they’re out there doing things*,* it shows*,* ‘OK*,* we’re willing to talk about it. We’re being transparent with what we’re doing.’ It immediately gets the community on your good side*,* that you’re not hiding away somewhere and we’re wondering what you’re up to.” – Interviewee 25 (community member)*.

Here, residents suggest the operators have been consistent and reliable in their engagement efforts, and that the community has tended to stay well-informed throughout the process. The resident cited above reminds us that a key driver of this approach is the operators’ engagement ethos – a commitment to honesty and transparency. In addition, it hints at another key aspect of the operators’ approachability and availability: they make sure to show up in popular spaces, places, and events within the community, instead of only being available through formal community information events. Overall, operators across all three sites described the importance of establishing engagement practices that create a culture of community embeddedness.

In one instance, this community embeddedness led to a local community energy group becoming an important supporter of the geothermal energy industry. One group member mentioned their support of the geothermal project and discussed their continued working relationship with the operator in the community:

*“We invited them [operator] to be a participant in our green energy and electric vehicle day*,* of which we have now had three*,* and they’ve come to every single one*,* and other events as well. Because [operator] are very active in reaching out to people and I think they found that…they do open days to which we always go*,* but actually having public events in the village hall is useful to them as well*,* because they’re meeting different people and they’re able to answer questions on a one-to-one basis.” – Interviewee 18 A (community member)*.

This provides an opportunity for local residents to become trusted authorities on a project, with enough distance from the direct benefits of it to be seen as more objective. Another approach that one operator used successfully is developing a community liaison group, which might include local community residents and local parish councillors. This creates opportunities for regular, direct communication between community residents and the operator, providing space for community input on the project throughout different stages of development.

#### Being proactive, responsive, and flexible

The planning requirements for engagement are very low, and there was much evidence in this research that operators routinely go well beyond them. They showed a proactive and continuous approach to engagement, not limited to the planning application stage. For instance, a local resident remembers how the local community was invited on site during drilling, and how the operator made specific efforts to engage children:

*“While they were in the major stage of drilling*,* they had*,* I think it was fortnightly drop-in sessions where you could go and meet some of the people and geologists there and just get it close to the rig really. They had a little viewing platform on top of their portacabins there and you could see what was going on. It was nice*,* I’d take the kids as well*,* they’re really welcoming and they’ve [got] colouring in sheets and things for the kids. So that was really nice and afterwards*,* the kids were like*,* ‘Oh*,* can we go there again? It was really good’.” – Participant 19 (community member)*.

Community engagement necessitates relationship-building with local community members and organisations to understand and address what the community wants. This can be done in small acts and day-to-day practices where an operator embodies the notion of being a ‘good neighbour’, such as sending hampers to show appreciation to community members, and “try and be quite nice” (Interviewee 16 – operator). This resonated with community members as being important, and as being demonstrated by the operators they had encountered:

*“I think they went out of their way to be good neighbours and the nearer you lived the more they engaged*,* so the people living down the road*,* constantly*,* ‘What are your concerns? What can we do about it?’ So they definitely went out of their way to be good neighbours.” – Interviewee 18B (community member)*.

Yet, it can also involve larger scale action like the establishment of a community fund to support organisations working on local issues (i.e., financial investments to serve bottom-up projects defined by the community):

*“[We] felt very much that we wanted to open it [the community fund] up so that it could be for anything that was gonna be of benefit to the local community…We’re not giving heat back to the local community so it’s about giving something back to the local community and that might be something that’s not directly environmentally related. We opened it up so it could basically be for anything…We had a couple of schools that wanted to create forest garden outdoor learning areas…We had one project from […] community trust which was funding the running costs of the mobile community larder for six months*,* so that’s basically a mobile food bank…that was really needed.…so it’s quite varied…So it’s [the community fund] a resource that hopefully that village*,* which has had to put up with most of any of the issues around construction…hopefully now they’ve got something permanent back that helps to recompense that.” – Interviewee 17 (operator)*.

We found that operator responsiveness, moving from active listening to addressing community concerns, showed good practice. This included being willing to invest time, money, and effort to mitigate impacts which concern community members. For example, one operator notes:

*“People there […] understood very well that drilling rig operating all night might be noisy and might be light pollution and there would be tankers going in and out and all the rest of it. So there were very tangible physical things that they were concerned about and we spent a lot of time and effort minimising those impacts and making people feel comfortable. it was OK. To our credit*,* when it transpired that we actually did this drilling*,* most people said*,* ‘Oh yeah*,* actually that wasn’t so bad*,* it was OK.’ So that was quite successful.” – Interviewee 3 (operator)*.

The operator continues, describing the willingness to use their financial resources to pro-actively reduce community impacts:

*“Right from the outset it was obvious that noise and traffic were the ones that were gonna bother people the most*,* noise by far the most worrisome*,* and we worked pretty hard on that. We picked the quietest rig we could*,* not the cheapest*,* we put sound mitigating measures around the site and we worked pretty hard at keeping the noise down. I think geothermal developers have to do that.” – Interviewee 3 (operator)*.

Being responsive also includes maintaining a flexible and adaptable approach to community engagement, which is especially important when working to address community dissatisfaction. One operator recalls a time they were confronted by a resident about insufficient engagement. They highlighted how they went about validating the resident’s assertions and acted to enhance their efforts for engagement to right these wrongs:

*“We got a very disgruntled letter from a resident…He was very much saying*,* ‘Look*,* this is all news to us. We don’t feel as though we’ve been consulted. We’ve got concerns about the nature of the project. There’s been seismicity at [anonymised].’ All the very legitimate things that a local resident who lives within a mile-and-a-half of the site might raise…My feeling was*,* right*,* we need to directly address what he’s saying because he probably represents a whole load of other people and I think he’s right. I suggested that we write a proper newsletter that detailed everything about the project and the timeline and then we distributed that to a much wider area. I put that together…and because of where we were with the pandemic and because of timing we couldn’t get it delivered for us so we literally hand-delivered it to*,* I think it was between 250 and 300 households*,* basically a good radius…because we thought those were potentially the people who were going to be concerned.” – Interviewee 17 (operator)*.

The same operator described how they listened to community feedback and acted to address it, noting the importance of being responsive and adaptable to practice good engagement:

*“It’s good to be responsive with the [engagement] strategy…We thought we’d got some things in place*,* it wasn’t enough*,* we responded directly to what people’s criticisms were. So they said*,* ‘You haven’t communicated with people far enough afield.’ We did it further afield. ‘We want more regular updates.’ We created new channels for updates. I think however good your plan is…there’s still potentially going to be ways that you can improve that plan in response to particular local requests. And so I think some degree of openness to that and willingness to adapt your approach and respond to*,* in many cases individuals*,* because individuals can be quite important […] in the local community [… is] really important.” – Interviewee 17 (operator)*.

Overall, this shows that operator’s commitment to honesty and transparency translated into being reflexive about their own practice and taking feedback from community members on board. This led them to being responsive and adaptable in their engagement practice.

## Discussion

### Identifying good practice

Research on engagement around energy infrastructure projects frequently shows community dissatisfaction with engagement efforts, where engagement is seen as a box-ticking exercise and economic incentives are perceived as bribes [44]. When communities are viewed as barriers and engagement as a statutory step in the planning process, operators tend to engage only to the degree necessary to reach these requirements. This is problematic because communities can interpret this as inauthentic, which can in turn feed community opposition to a project. Here, we tell a different story, highlighting how effective community engagement can be designed when operators’ views and approaches to communities and engagement are more positive. We found that when guided by an ethos that values communities and demonstrates an ethics of care, operators can establish meaningful community engagement practices based on early and consistent two-way information sharing, honesty and openness, commitments to trust-building, and responsiveness to local, place-based community needs.

Our research demonstrates the importance of involving non-scientists in project communications. It shows that trust-building is as valuable as science when it comes to engagement. This aligns with existing research on communication about underground energy interventions, according to which communication is often not about matters of “scientific” fact, but matters of community concern [63]. How community engagement is designed depends largely on *who* is designing it, and what sort of motivations and rationales are driving the engagement effort [23, 38, 64, 65]. Thus, involving local workers, the project team, and non-technical experts with engagement experience contributes to attitudes toward community members that are guided by increased understanding and empathy with communities and their concerns. Through committed relationship and trust-building practices, operators can better spot the residents’ major concerns, and design and adapt their engagement strategies to align with this.

While most operators are indeed striving to develop a technically successful project, this alone is not a sufficient driver for the establishment of good community engagement practices, as communities are merely seen as obstacles to project goals. How operators perceive communities and how they see themselves in relation to them (e.g., as part of, rather than separate from, the community) is crucial. Where an engagement ethos is characterised by a sense of relational obligation toward a community, community members are not demonised by industry actors but engaged from a perspective of empathy and understanding. A relational engagement ethos means that operators value spending time and resources on engagement, denoting an ethics of care [66–68]. This ethos shapes the way good engagement practices are designed and established. An emphasis on engagement practices derived from an ethics of care supports the broader agenda of developing and adhering to “geoethics” [69] in the industry more widely.

Owing to the emergent nature of the technology in the UK, evidence of the extent to which geothermal projects benefit local communities is currently limited to a few projects. Abesser et al. [7] provide some examples for the UK where geothermal projects have contributed to local jobs and the local economy across a range of sectors. Other benefits such as local heat (or electricity) provision or opportunities for financial investments (e.g., for co-ownership) could be plausible in the future but have not yet been observed. Certainly, the local operators interviewed as part of this research showed that they are taking local benefits seriously and are actively looking for diverse ways to provide them.

Deep geothermal projects in England currently require planning permission from Local Planning Authorities (LPAs). As part of the permissioning process, LPAs are required by law to consult the public on the proposed development [70]. This is usually done by the LPA through inviting submissions via a web portal; a type of engagement that is one-way and instrumental in nature. There is no legal requirement for developers and operators to carry out public consultations or engagement. However, the operators who took part in this research recognised the benefits of it and are routinely doing so. This could be better supported by a national policy framework that mandates meaningful engagement processes before and during the planning and implementation of projects (e.g., including deliberation about a potential project, before the proposal stage). This could go hand-in-hand with acknowledging that engagement needs to be resourced properly throughout the lifetime of a project. This would signal a ‘culture change’ [26] to communities, demonstrating that their interests are foregrounded. Our research indicates that operators would be able to take this on board and successfully balance commercial and community interests.

An important question that was beyond the scope of our research, but warrants further investigation, concerns the funding of community engagement. While the operators involved in our study are already committing time and financial resources to engagement activities, such efforts can add significant costs to projects that often operate within tight financial margins. Community engagement should be a core component of responsible and sustainable development, and it could be argued that the primary beneficiaries of geothermal developments—namely, the companies that gain economically—have a responsibility to invest in meaningful engagement with affected communities. This parallels the principle of environmental impact assessments (EIA), where the onus is placed on the scheme operator to assess and mitigate potential negative impacts on the environment. Nevertheless, strategic support from public authorities—such as local planning authorities or other relevant regulators—can play an important role in improving the overall acceptance, inclusiveness, and effectiveness of engagement. Importantly, the financial implications of such requirements for developers and operators must be recognised and acknowledged, particularly given the constrained budgets typical of geothermal projects. We recommend that future research further explores this question of funding for geothermal as well as other large renewable projects, including international examples of good practice from beyond the UK.

Another suggestion for improvement is enhanced training in engagement practices for staff members for two reasons: (1) it would encourage the hosting of community engagement processes by operators themselves (instead of relying on marketing and PR firms) and facilitate engagement tailored to the local and historical context of projects [23]; and (2) it would build expertise in engagement, allowing operators to focus on trust building, incorporating public input and deepening relationships rather than on public image.

### Limitations

There are some caveats and limitations to our findings. First, the research was conducted with a small number of actors across three sites. As such, we cannot make generalised statements about large-scale community trust in operators. Indeed, one interviewee suggested that it can be difficult to gain a comprehensive overview of the potential risks because an operator is “in it for the business.” In addition, several interviewees expressed that the follow-ups they were expecting were not always materialising, and raised that the COVID19 pandemic had created challenges for engagement. Yet, as restrictions were lifting when we interviewed some of the participants, they explained that they had expected engagement to pick up again more quickly than it did. Still, overall, the local community members interviewed expressed trust in the geothermal operators and supported the existing projects, and what made the operators’ attitudes and approaches to engagement stand out is that the desire to move a project forward successfully was not the *only* driver for excelling at community engagement.

However, the UK geothermal landscape is not without community contestation. One operator recently proposed several new geothermal sites, which have been met with some resistance, both in terms of ‘place-fit’ issues [48] and a lack of sufficient engagement, further demonstrating the importance of ensuring that thorough community engagement is developed for each project, even when building on a previously successful project. Still, being able to acknowledge and address mistakes around community engagement can create opportunities for honesty, accountability, and trust building. Finally, there were some occasions where operators lamented a lack of public interest in geothermal energy. This suggests that some myths about communities may persist in operator circles. Despite these caveats, we believe that the current UK geothermal sector provides positive examples of how good practices around community engagement can form and persist throughout the course of a project lifecycle.

## Conclusions

This article explores perceptions and experiences of community engagement across the nascent UK geothermal sector to tease out what meaningful community engagement practice looks like. We have shown how the attitudes and values of operator engagement actors shape the design and pursuit of community engagement efforts. We found that (1) having a plurality of engagement actors can be useful for designing meaningful engagement practices; (2) engagement leaders’ attitudes toward community members emphasised respect, empathy and honesty, with value placed on trust- and relationship-building; and (3) this engagement ethos translated into the design of engagement activities that created space for the communities to work with the operators. Many of the community members interviewed expressed general support for the positive attitudes of the operators and their way of conducting engagement. Overall, we have demonstrated the importance of the operators’ ethos in shaping community engagement. Our paper contributes to existing research by providing some of the first understandings of community perceptions of geothermal energy projects in the UK.

There is an urgent need for the geothermal energy sector, in collaboration with planners, regulators and academics, to develop a comprehensive public engagement framework, which policymakers can contribute to, given the direct connections between community engagement, public support, and policy decisions [71]. This is particularly important for underground energy interventions that might involve risk concerning the potential unknowns and uncertainties tied to subsurface activity. Given the degree to which community-supported energy projects may be more successful, the UK’s ability to decarbonise the energy sector and move toward net zero will depend in part on the degree to which renewable energy projects can continue to work with communities to move forward. There is a practical need to enhance community engagement practices for future renewable energy projects, to ensure an energy transition that is both sustainable and just. We acknowledge that not all practices will be transferable across technologies; yet many of the principles outlined are not technology-specific and broad enough to be useful to other projects. Future work will need to deepen our understanding of public perceptions of deep geothermal energy, explore further geothermal-specific challenges such as uncertainty in engagement, and take a more longitudinal approach than we were able to, owing to the nascent state of the industry in the UK.

## Supplementary Information

Supplementary Material 1

## Data Availability

The data is not available at the time of submission of publication due to ethical restrictions regarding anonymity.

## Footnotes

1 The term ‘operator’ includes stakeholders that are ‘developers’; in some projects, developers are different from operators. For ease of reading, we use ‘operator’ as a shorthand for operators and/or developers.
