# Supplementary material for: Operators’ good community engagement practices in energy projects: examples from the UK deep geothermal sector
Source: Energy Sustain Soc. 2026 Feb 19;16(1):19. doi: 10.1186/s13705-026-00566-y (PMC13048911; doi:10.1186/s13705-026-00566-y)
Supplement: Supplementary file 1 — Supplementary Material 1 [file 13705_2026_566_MOESM1_ESM.docx]

# Underground energy on the ground

## Interview guide for Operators

1. Please provide a little background on yourself, where you’re from, your age, education, family. Do you have any connections to [location]?
2. Tell me about your position at (operator). How long have you worked here, what is your job, what did you do before this? In this position, what is your role specifically in the case of the geothermal project at [location]?
3. Walk me through the process of site development. When did it begin and where are things at now?
4. Who do you see as key stakeholders when it comes to making decisions about this proposed geothermal site?
5. What individuals or organizations have you worked with on decision-making about the proposed geothermal project?
   1. Activists? Local or national government? Industry?
   2. Who has potentially not been involved but perhaps should have been?
6. In the case of this proposal, who are the residents or community members that should be engaged by the developer and/or the local government authority? Who should have a stake in and be able to influence this process as a community member?
7. What has been your organization’s approach to engaging the public on this proposed project?
8. What are the main factors in choosing how to engage the public on this issue?
9. What feedback have you gotten from local residents, organizations, business owners or local government on how this strategy has been received?

1. In an ideal world, what would your community engagement efforts for this project look like and who would be involved?
2. What are the most prominent or common concerns that the public has expressed about the project? The local council?
3. What has the working relationship with the local council looked like? How has the local council approached community engagement around this project?
4. What are the potential benefits or risks and concerns you see to the proposed geothermal energy project?
   1. What sort of concerns do you have about how this proposed geothermal project could impact people or places in this area?
   2. Are potential risks, impacts or benefits evenly distributed?
   3. ‘Who and where do you think will be directly impacted by the proposed geothermal project?
   4. How far away from the proposed site do you think these impacts will extend?
5. Is community engagement on this process necessary?
6. What constitutes good community engagement?
   1. To what degree might community members benefit directly from this project, i.e. compensation, ownership, access to heat, etc. What benefits do you think community members would like to see?
7. To what degree might community members benefit directly from this project, i.e. compensation, ownership, access to heat, etc. What benefits do you think community members would like to see?
8. Where and how can we enhance spaces for different stakeholders to participate?
9. Is there anything specifically about this place makes it suitable or unsuitable for geothermal energy?
   1. Alternative locations or places where you think this activity you think could be acceptable/unacceptable?
   2. Would you feel differently about other energy projects such as wind or solar farms?
10. What level of governance do you think is best suited to granting consent or refusal or regulate geothermal sites? Local level (e.g., council planning authority), National level (England, Scotland, Wales, Northern Ireland), or UK level (e.g., Secretary of State)
11. What potential benefits or risks do you see tied to other types of energy development, specifically shale gas?
    1. Induced seismicity?
12. Do you have any similar concerns about geothermal?
    1. Potential induced seismicity tied to geothermal energy development and storage?
    2. Do you think any risk can be successfully mitigated through regulation?
    3. How do you think it should be regulated?
13. How might this geothermal project relate to the impact of climate change?
14. Do you have any other thoughts on the geothermal project or geothermal energy in general, or is there anything we may have missed?
15. Who else would be important people for me to talk to about this issue?

## Interview guide for residents

**Background Questions**

1. Please provide a little background on yourself, your family, and your daily life here [location]
   1. How long have you lived here? Born here? Family here?
   2. In a given year, about how much of your time is spent here versus elsewhere?
   3. Age? Occupation? Do you work locally?
2. How would you describe your local area?
3. What, if any, historical connections does this area have to different energy industries?
   1. What type of energy activities?
   2. Any personal connections to these (I.e. selves, friends, family)?

**Knowledge & Support for Local Geothermal Project**

1. What do you know, if anything, about geothermal energy in general?
2. What do you know, if anything, about local plans for geothermal energy development?
3. (If previously aware) How and when did you first hear about the project?
4. What, if any, potential local benefits might there be from the proposed geothermal energy project?
   1. What local benefits would you like to see?
   2. Who do you think could potentially benefit from the proposed geothermal project?
5. What, if any, potential risks or concerns might you have about the local impacts of the proposed geothermal energy project?
   1. Who and what areas do you think could potentially be impacted by the proposed geothermal project?
   2. How would you like to see these concerns addressed?
6. Is there anything specifically about this place that makes it suitable or unsuitable for geothermal energy?
   1. Alternative locations or places where you think this activity you think could be acceptable/unacceptable?

**Operator community engagement efforts & residents’ experiences & expectations**

1. Is community engagement on this process necessary?
2. What would constitute good community engagement for this geothermal project?
   1. To what degree might you or other local residents benefit directly from this project, i.e. compensation, ownership, access to heat, etc. What benefits do you think community members would like to see?
3. What has your experience of community engagement for this project looked like?
   1. Are you aware of any efforts from [operator] to inform or consult local residents about this project? Local government efforts? Any community organizations?
4. Do you feel that your views on the geothermal project are being listened to? Will they be taken into account by the developer or the council?
   1. If so, how? If not, why not?
   2. Whose views are being taken into account, and whose views should be?
   3. Who else is involved or should be able to have a say or influence the decision on this proposal? Why?

**Concluding Questions**

1. Do you have any other thoughts on the geothermal project or geothermal energy in general, or is there anything we may have missed?
2. Who else would be important people for me to talk to about this issue?
